# Supplementary material for: Linear magnetoelastic coupling and magnetic phase diagrams of the buckled-kagomé antiferromagnet Cu3Bi(SeO3)2O2Cl
Source: Sci Rep. 2022 May 5;12:7383. doi: 10.1038/s41598-022-11368-5 (PMC9072401; doi:10.1038/s41598-022-11368-5)
Supplement: Supplementary file 1 — Supplementary Information. [file 41598_2022_11368_MOESM1_ESM.pdf]

Supplemental Material: Linear magnetoelastic coupling and magnetic phase diagrams of the buckled-kagomé antiferromagnet $Cu_3Bi(SeO_3)_2O_2Cl$

S. Spachmann[1,*], P. Berdonosov[2,3], M. Markina[2], A. Vasiliev[2,3], and R. Klingeler[1]
[1]*Kirchhoff Institute for Physics, Heidelberg University, D-69120 Heidelberg, Germany*
[2]*Lomonosov Moscow State University, Moscow 119991, Russia and*
[3]*National University of Science and Technology "MISiS", Moscow 119049, Russia*
(Dated: April 19, 2022)

I. SAMPLE ORIENTATION

Laue x-ray diffraction (XRD) and angle-dependent magnetization measurements at $T = 2$ K and $B = 5$ T are shown in Fig. S1. Laue XRD data were used to orient the samples precisely for in-plane thermal expansion along the a and b axis, whereas the angle-dependent magnetization (Fig. S1(d)) served to orient the samples for magnetization measurements along the easy (b axis) and hard (a axis) in-plane directions.

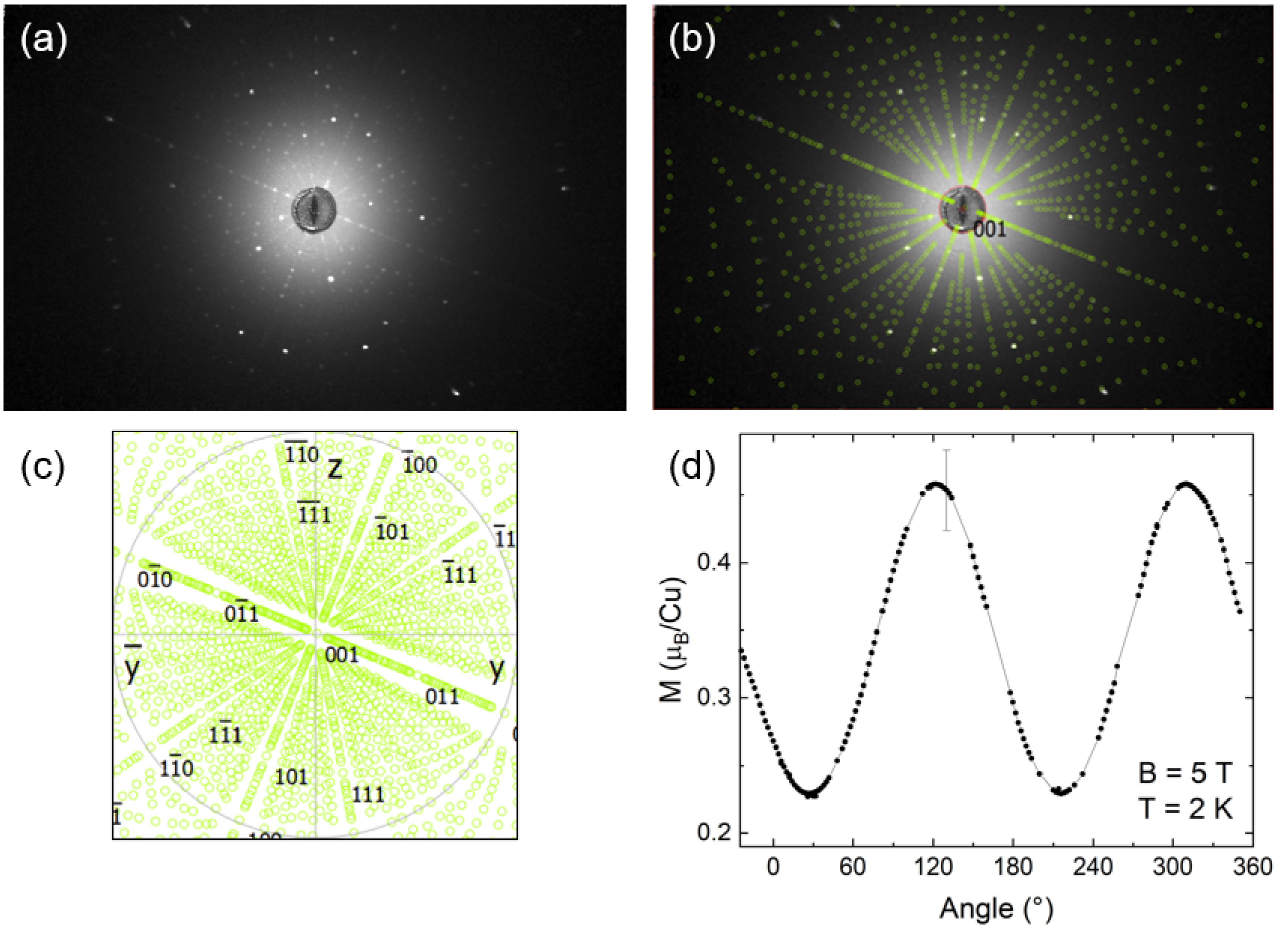

Figure S1: Orienting the samples: (a) Exemplary Laue XRD image and (b) corresponding orientation depicted in the Laue image itself and (c) a wider stereographic projection. (d) In-plane angular dependence of the magnetization at 2 K and 5 T. The error bar is representative for all data points and mainly results from mass determination.

II. LATTICE AND COUPLINGS

The lattice of the francisite $Cu_3Bi(SeO_3)_2O_2Cl$ according to Ref. 1 along with the dominant couplings is presented in Fig. S2.

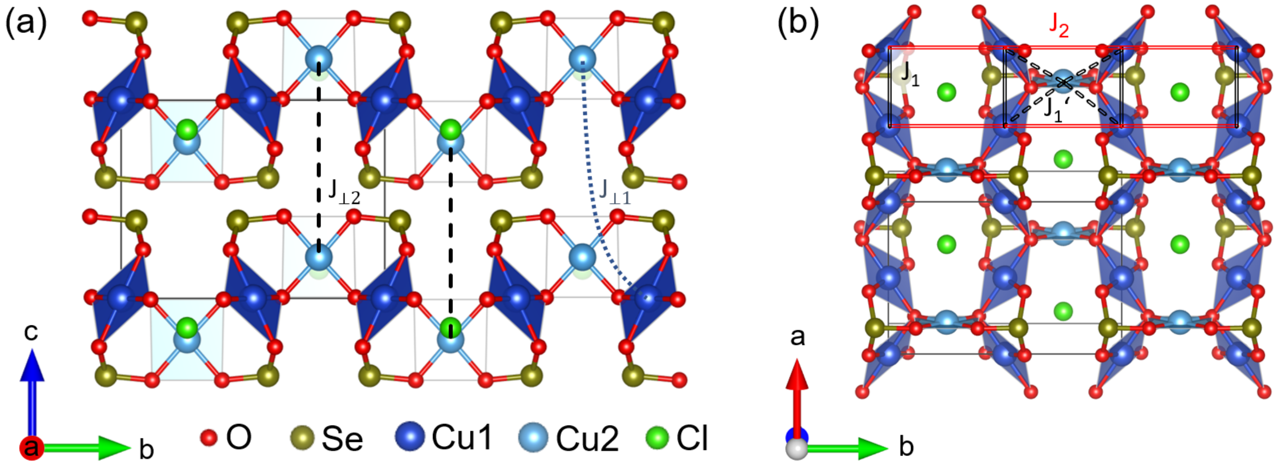

Figure S2: Crystal structure of $Cu_3Bi(SeO_3)_2O_2Cl$ for (a) the *bc*-plane and (b) the *ab*-plane. Thin black lines mark the non-magnetic high temperature orthorhombic *Pmmn* unit cell. Black, red and blue lines mark different exchange couplings. Bi ions are omitted for clarity and (b) is slightly tilted forward for better visibility. Visualization by VESTA.[2]

III. ADDITIONAL THERMAL EXPANSION DATA

A. Volume Thermal Expansion

The volume thermal expansion, gained from summing over the a, b, and c axis, is shown in Fig. S3. A strong kink is seen in dV/V at T_{N} (Fig. S3(b)), corresponding to a positive λ-like anomaly in the volume expansion coefficient β (Fig. S3(a)). At T_{S} a jump in β occurs. The additional peak is most likely an artifact from summing the three linear thermal expansion coefficients of the a, b, and c axis to obtain the volume expansion.

B. Phononic Background Fits

Phononic background fits to the thermal expansion coefficients and the specific heat are shown in Fig. S4. Both thermal expansion and specific heat are fitted by Debye and Einstein contributions according to

$$c_p^{ph} = n_{\mathrm{D1}} D\left(\frac{T}{\Theta_{D1}}\right) + n_{\mathrm{D2}} D\left(\frac{T}{\Theta_{D2}}\right) + n_{\mathrm{E}} E\left(\frac{T}{\Theta_{E}}\right), \qquad (1)$$

where $n_{\mathrm{D1,2}}$ and n_{E} are constants, and $D(T/\Theta_{\mathrm{D1,2}})$ and $E(T/\Theta_{\mathrm{E}})$ are the Debye and Einstein functions with the Debye and Einstein temperatures $\Theta_{\mathrm{D1,2}}$ and Θ_{E}. A fit to the specific heat (Fig. S4(a)) below the onset of T_{S}, at 35 K to 93 K, yields $\mathrm{n_{D1}} = 3.25$, $\mathrm{n_{D2}} = 6.14$ and $\mathrm{n_E} = 12.1$ with $\Theta_{\mathrm{D1}} = 127$ K, $\Theta_{\mathrm{D2}} = 377$ K and $\Theta_{\mathrm{E}} = 1063$ K. These Debye and Einstein temperatures are then used to fit the thermal expansion data in the range from 35 K to 60 K. Due to the high value of the Einstein temperature, however, the contribution of Einstein modes to the thermal expansion below 100 K is negligible. Therefore, and in order to reduce the number of free parameters, the Einstein mode is omitted for the thermal expansion fit. The resulting background fits are shown in Fig. S4(b). The fits for the c axis and volume describe the data very well – the c axis up to about 80 K and the volume up to T_{S} – while the a and b axis only coincide with the data in a narrow temperature range, roughly between 40 K and 54 K.

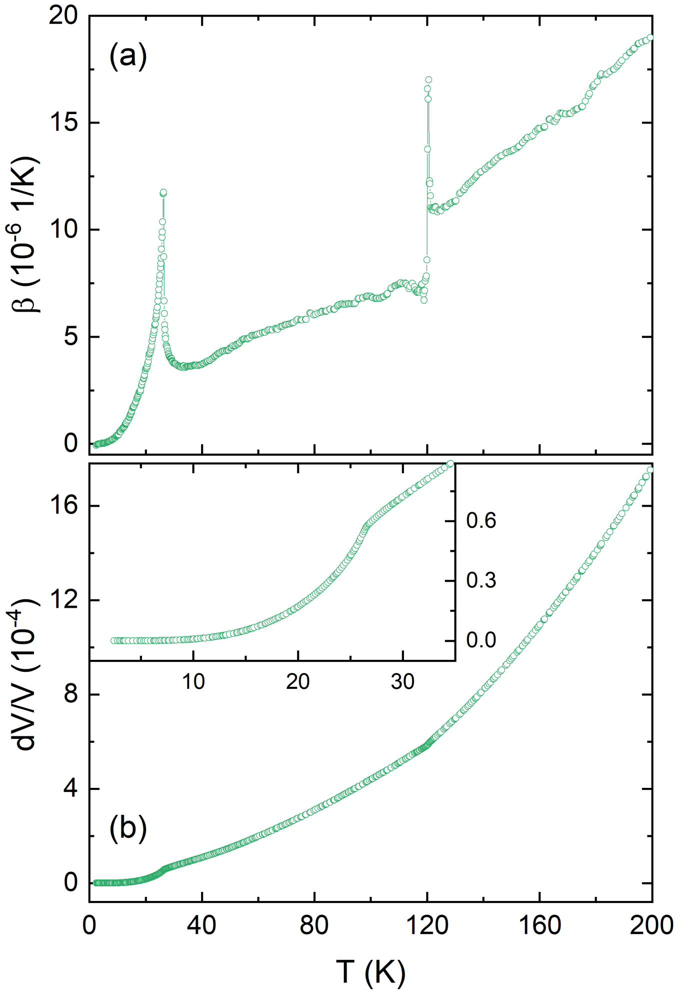

Figure S3: (a) Volume thermal expansion coefficient β and (b) volume changes dV/V in zero-field, calculated by adding α_i, i $= a$, b, c, and subsequent integration to obtain dV/V. The inset in (b) shows a magnification of the low temperature window.

C. Extraction of Jump Heights

The extraction of jump heights at $T_{\rm S}$ by an area-conserving (and entropy-conserving for $c_{\rm p}$) method for the thermal expansion and specific heat is shown in Fig. S5. Jump heights are indicated in the figure.

D. Grüneisen Ratios

Fig. S6 shows the magnetic Grüneisen ratios $\Gamma_{i,\rm mag} = \alpha_{i,\rm mag}/c_{\rm p,mag}$ for all axes and the volume. Notably, the $\Gamma_{i,\rm mag}$ reach a plateau around $T_{\rm N}$ (26.4 K) down to about 22 K, then vary upon cooling and reach another plateau below 15 K. The changes between 15 K and 22 K indicate the evolution of a competing energy scale in this regime.

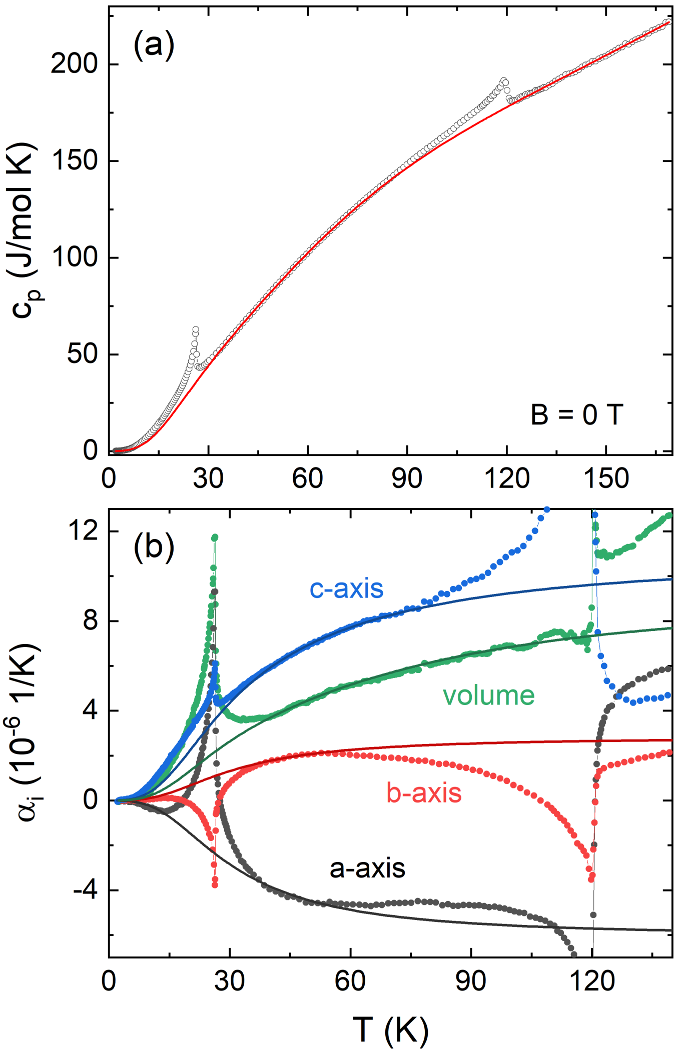

Figure S4: Phononic background fits (lines) to (a) the specific heat and (b) the low temperature thermal expansion data (circles) in zero-field as explained in the text.

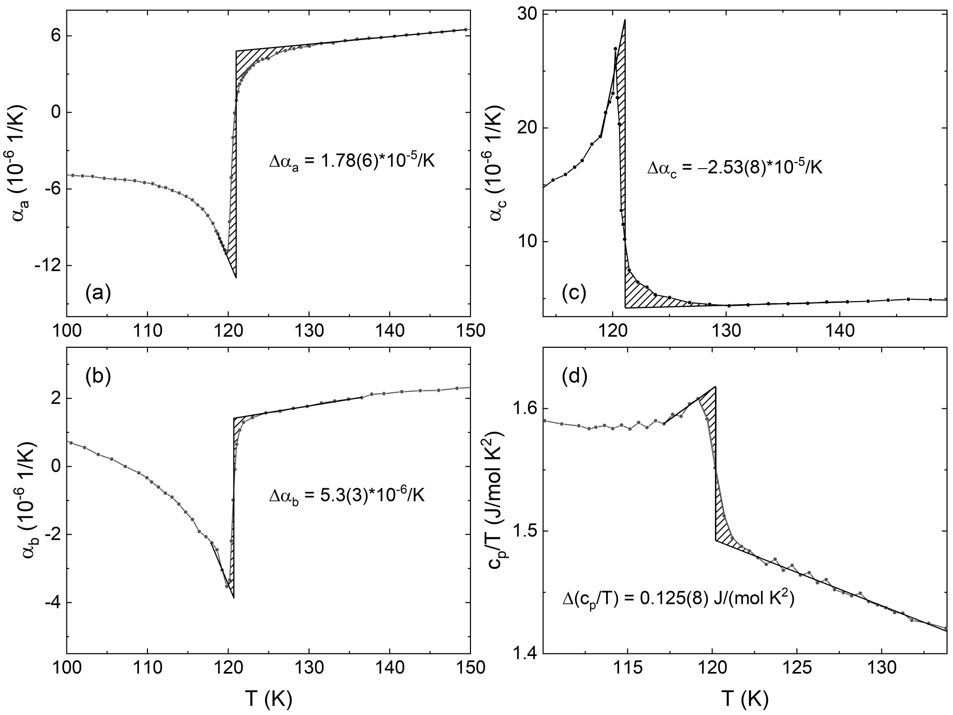

Figure S5: Determination of the jump heights in (a-c) thermal expansion and (d) specific heat by an area-conserving method.

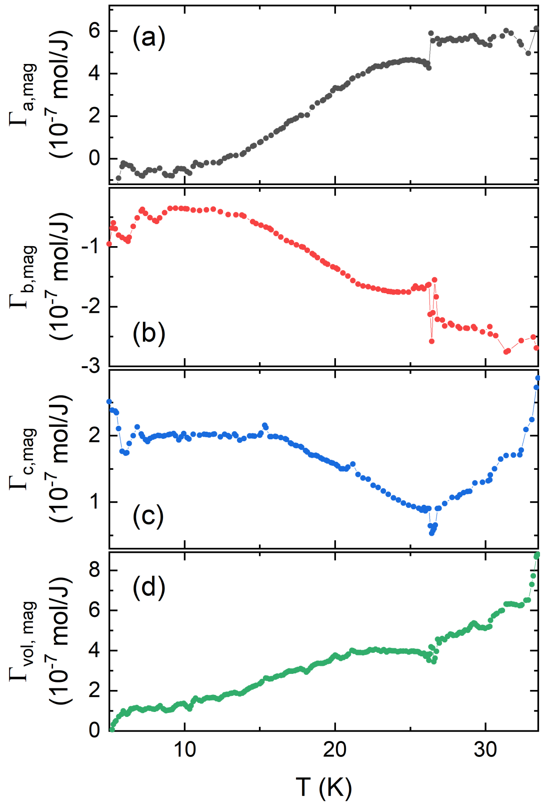

Figure S6: Magnetic Grüneisen ratios $\Gamma_{\mathrm{mag},i} = \alpha_{\mathrm{mag},i}/c_{\mathrm{p,mag}}$ for (a) a, (b) b, and (c) c axis, as well as for (d) the volume.

E. Thermal Expansion at 0 T and 15 T

A comparison of thermal expansion data at 0 T and 15 T, normalized above T_S, shows in-plane field effects up to about 100 K as described in the main article. (Fig. S7).

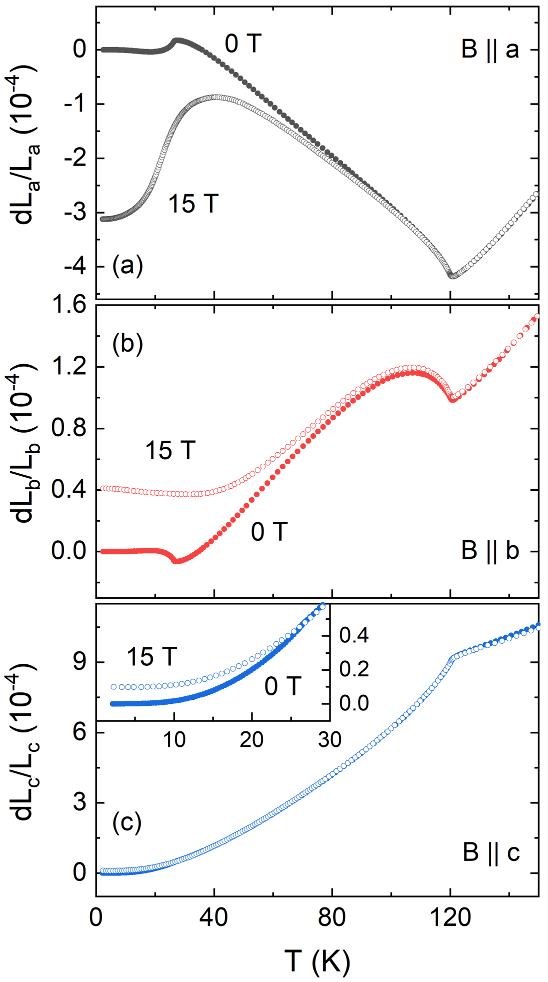

Figure S7: Comparison of relative length changes dL_i/L_i in zero-field (closed circles) and B = 15 T (open circles). Inset in (c) shows a magnification of the low temperature window.

IV. FAILURE OF GRÜNEISEN SCALING BELOW T_N

In the main text we mentioned the failure of Grüneisen scaling below 22 K (see Fig. 2). Here, we want to spend some more time to explore this behavior. Fig. S8 shows the magnetic contributions to the specific heat and the thermal expansion in zero-field and at $B > 0$ up to 7 T and 15 T, respectively. The same phononic background as described in the main text was used for all data sets. For better comparability with $c_{\mathrm{p,mag}}/T$ we plotted $\alpha_{\mathrm{c,mag}}/T$ in Fig. S8(b). The specific heat data shows two anomalies, one at 15 K which is not affected my the magnetic field and one which shifts to higher temperatures and broadens as the field is increased. The latter marks the crossover from the field-induced ferrimagnetic phase to the paramagnetic phase. Similarly, $\alpha_{\mathrm{c,mag}}/T$ shows a peak at 15 K which is insensitive to a magnetic field and an additional shoulder may be seen around 30 K at 1 T, which is indistinguishable from the high-temperature tail of the 15 K peak at 15 T. So what is the origin of the peak at 15 K? A Schottky anomaly can be ruled out, because in contrast to the observed behavior it would shift in an applied magnetic field unless it were from transitions of electrons between energy levels of the same spin quantum number m_S. Plotting $\alpha_{\mathrm{c,mag}}/T^2$ vs. T (Fig. S8(b) inset) shows a linear rise up to about 10 K, i.e., $\alpha_{\mathrm{c,mag}} \propto T^3$, with a negative offset at $B > 0$. While the offset can be related to ferromagnetic magnons – $\alpha_{\mathrm{FM}} \propto c_{\mathrm{p,FM}} \propto T^{3/2}$ – the linear behavior signals

either phononic or antiferromagnetic (AFM) magnonic contributions. In the ferromagnetic phase above the critical field no AFM magnons can be present, therefore the linear rise suggests a phononic origin of the 15 K anomaly judging from the thermal expansion data. In contrast, the rise of $c_{\mathrm{p,mag}}/T^2$ (inset of Fig. S8(a)) is not as clearly linear and additional effects which do not show up in the thermal expansion may be present, leading to the failure of Grüneisen scaling.

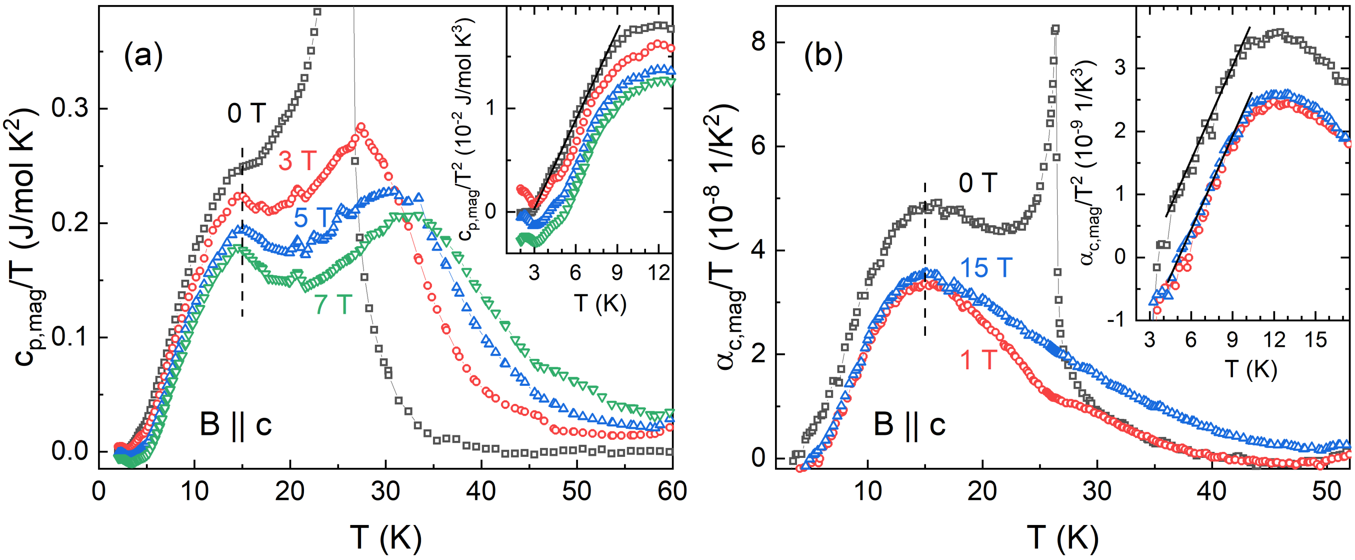

Figure S8: Magnetic contributions to (a) the specific c_p/T and (b) the thermal expansion α_c/T at zero-field and in fields $B \parallel c > 0$ as shown in the figure. Vertical dashed lines mark the peak position at 15 K. The insets show $c_{\mathrm{p,mag}}/T^2$ and $\alpha_{\mathrm{c,mag}}/T^2$ vs. T. Black lines are guides to the eye.

Considering results from other experimental techniques, there have been several observations reported in the AFM and FM phases for $B \parallel c$ in $Cu_3Bi(SeO_3)_2O_2Cl$ which may be related to the anomaly: Infrared reflection and transmission studies found two low-frequency phonon excitations at 5 K.[3] One of them, at 33.1 cm^{-1} ($\approx$ 48 K), is visible in zero-field and only shifts slightly, to about 35 cm^{-1} in fields $B \parallel c$ of 10 T. For $B \perp c$ the resonance frequency of this phonon mode decreases. This phonon branch was tentatively assigned to magnon excitations[3], based on an analysis of previously reported oscillator strengths of magnons and electromagnons. The second phonon excitation at 5 K was only observed for $B \parallel c$ in the ferromagnetic phase, with a frequency of 10.5 cm^{-1} ($\approx$ 15.1 K) at 1 T.[3] This phonon, however, shifts linearly in an applied magnetic field, up to about 16 cm^{-1} (23 K) at 7 T.

Furthermore, a global spin gap of 1.57 meV (18.2 K) was reported from inelastic neutron scattering experiments.[4] This fits with the temperature scale of the anomaly and would suggest a relation to magnonic excitations. The presence of the anomaly in zero-field, i.e., in the AFM phase, would then be explained by ferromagnetic spin-waves within the ferromagnetically coupled layers.

Lastly, two resonances at 1.23 meV (14.3 K) and 1.28 meV (14.8 K) were observed in the brother compound $Cu_3Bi(SeO_3)_2O_2Br$ in time-domain THz spectra at 3.9 K.[5] Electron spin resonance (ESR) measurements from the same report suggest that these two resonances are of magnetic origin and shift to higher frequencies in higher magnetic fields. However, a flat, i.e., field-independent, resonance would not be seen by spectroscopic field-sweeps around 300 GHz. Frequency sweeps at different magnetic fields would be necessary to observe such a resonance.

In conclusion, low-energy optical phonons, potentially coupled to magnon excitations, seem to be the cause for the anomalies observed around 15 K.

V. MAGNETIZATION MEASUREMENTS

The isothermal magnetization at 2 K up to 7 T as well as the static magnetic susceptibility at 1 T are shown in Fig. S9. Measurements of the isothermal magnetization up to 14 T at temperatures up to 25 K (a and b axis) and up to 50 K (c axis) are shown in Fig. S10. Phase boundaries for the phase diagrams (Fig. 6) were obtained from the peaks in the magnetic susceptibility $\partial M_i/\partial B$. The static magnetic susceptibility for all axes between 1 T and 14 T is shown together with the Fisher specific heat in Fig. S11. Phase boundaries for the phase diagrams in Fig. 6 were obtained from the peaks in the Fisher specific heat for the in-plane directions and the c axis up to 0.5 T. Above

0.5 T phase boundaries were obtained from the temperature at half of the jump heights upon entering and exiting the intermediate mixed phase. Table S1 shows the experimental results from our magnetization measurements in comparison to the results of calculations by Nikolaev et al.[6]

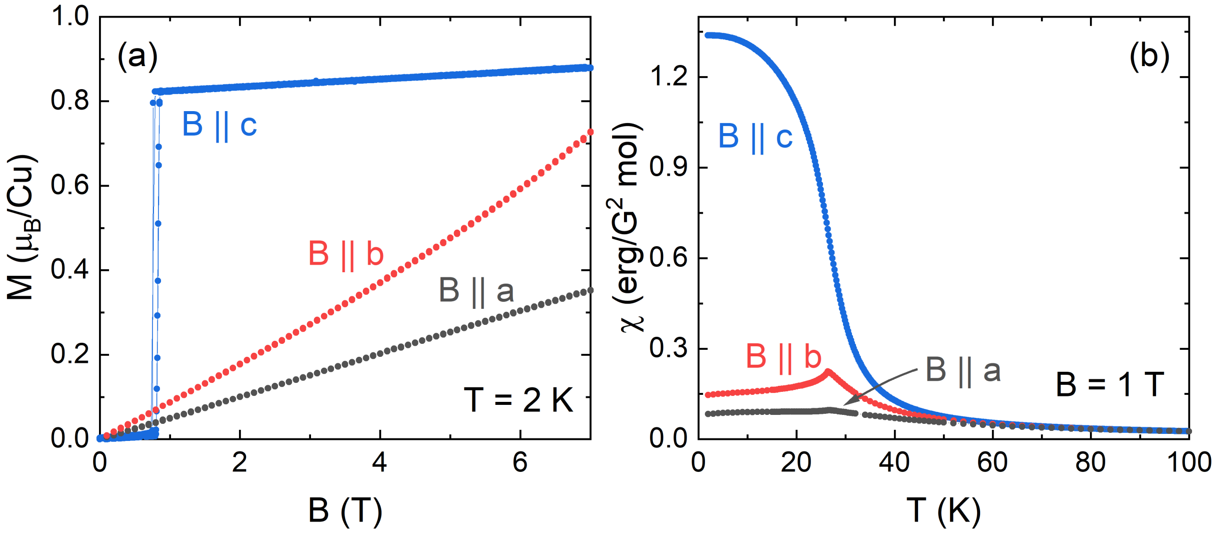

Figure S9: (a) Isothermal magnetization at T = 2 K and (b) static magnetic susceptibility $\chi = M/H$ at $B = 1$ T for $B \parallel a$ (black), $B \parallel b$ (red), and $B \parallel c$ (blue). Error bars are on the order of the size of the data points.

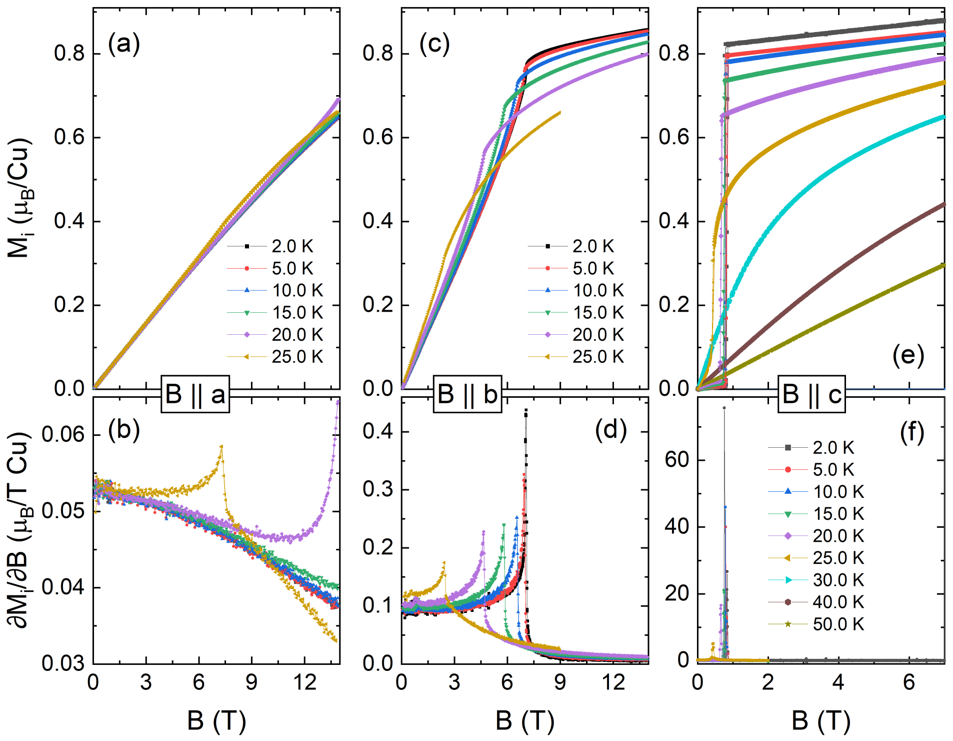

Figure S10: Isothermal magnetization M_i and magnetic susceptibility $\partial M_i/\partial B$ at low temperatures for (a, b) $B \parallel a$, (c, d) $B \parallel b$ and (e, f) $B \parallel c$.

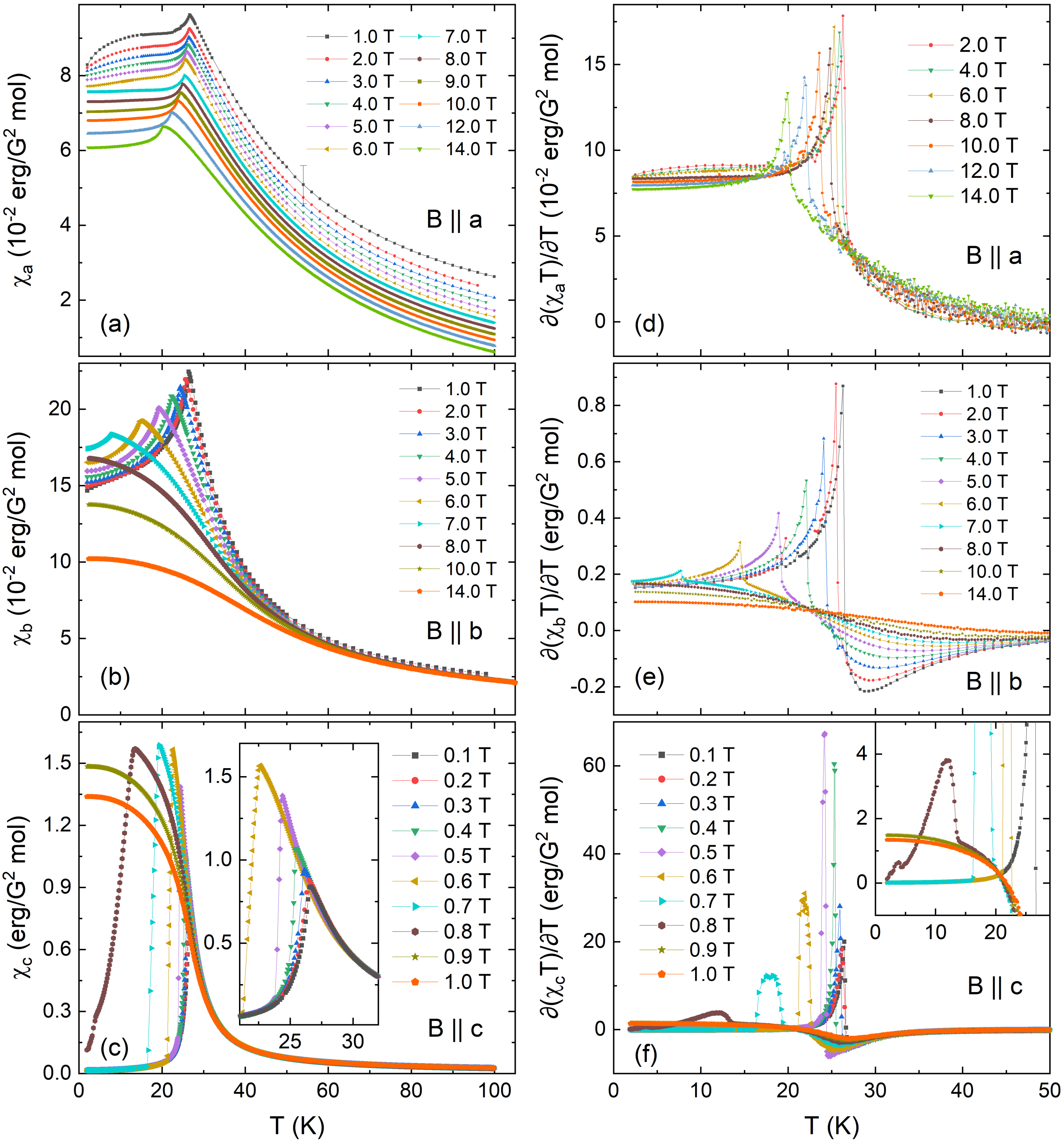

Figure S11: Static magnetic susceptibility $\chi = M/H$ and derived Fisher's specific heat in magnetic fields up to 7 T for $B \parallel a$ (a, d), $B \parallel b$ (b, e), and $B \parallel c$ (c, f). The inset in (c) shows a magnification for fields up to 0.6 T around T_N. The inset in (f) shows a magnification of the low temperature region. Data in (a) are offset by $-1.5 \cdot 10^{-3}$ erg/(G^2 mol).

Table S1: Experimental results from our magnetization measurements in comparison to the results by Nikolaev et al.[6]

	$\partial M_a/\partial B$ (μ_B/T)	$\partial M_b/\partial B$ (μ_B/T)	$\partial M_c/\partial B$ (μ_B/T)	W_b (μ_BT)
This work	0.045(3)	0.089(3)	0.009(2)	2.26
LDA+SO[6]	0.15	-	0.0073	-
Hartree Fock[6]	0.044	-	0.0061	2.2

VI. QUANTITATIVE ANALYSIS OF THE PHASE BOUNDARIES FOR B || c

Table S2 shows the quantities extracted from magnetization and dilatometry measurements upon sweeping the magnetic field (B) or the temperature (T) and the resulting calculated quantities obtained according to Eq. (2) to (4) in the main text.

Table S2: Jump heights, calculated changes in entropy, as well as field and pressure dependencies for the discontinuous transition in magnetic field from AFM to FM for $B \parallel c$ according to equations (2) to (4). The quantity $\partial B_{\mathrm{crit}}/\partial T$ was calculated by taking the derivative of two polynomial fits in different temperature regimes to the values $B_{\mathrm{crit}}(T)$ in the phase diagram.

AFM → FM – c axis: Extracted and calculated quantities									
T (K)	B_{crit} (T)	Sweep	$\Delta L_c/L_c$ (10^{-6})	ΔM (μ_B/Cu)	$\partial B_{\mathrm{crit}}/\partial T$ (mT/K)	ΔS_{calc} (mJ/mol K)	$\partial T_{\mathrm{crit}}/\partial p_c$ (K/GPa)	$\partial B_{\mathrm{crit}}/\partial p_c$ (mT/GPa)	$\partial \ln(B_{\mathrm{crit}})/\partial p_c$ (%/GPa)
2.0±0.1	0.82	B	7.8±0.5	0.82±0.02	–2.2±1.0	30±14	34±16	76±6	9.3±0.7
5.0±0.1	0.81	B	7.8±0.5	0.8±0.02	–2.2±1.0	29±14	35±17	78±6	9.6±0.7
10.0±0.2	0.80	B	7.5±0.5	0.78±0.02	–7±3	90±40	11±5	77±6	9.6±0.7
15.0±0.2	0.75	B	7.2±0.5	0.74±0.02	–13±3	160±40	6.0±1.5	77±6	10.4±0.8
17.9±0.4	0.70	T	6.8±0.5	0.69±0.07	–17±3	200±40	4.6±1.0	79±10	11.3±1.4
20.0±0.2	0.66	B	5.8±0.4	0.65±0.02	–27±8	290±90	2.6±0.8	71±6	10.8±0.9
21.9±0.3	0.60	T	5.1±0.4	0.56±0.06	–38±8	360±90	1.9±0.5	72±10	12.1±1.6
24.0±0.2	0.50	T	3.1±0.4	0.36±0.04	–60±20	360±130	1.1±0.5	69±12	14±3
25.0±0.2	0.44	B	3.1±0.4	0.20±0.02	–94±20	320±80	1.3±0.4	120±20	28±5
25.3±0.2	0.40	T	1.2±0.3	0.12±0.04	–142±30	280±120	0.6±0.3	80±40	20±9

VII. CRITICAL SCALING ANALYSIS

The magnetic contributions to the thermal expansion coefficients and the specific heat in Fig. 7 in the main text were fitted by Eq. (5), i.e.,

$$c_\mathrm{p} = \frac{A^\pm}{\alpha^\pm}|t|^{-\alpha^\pm}(1 + E^\pm|t|^{0.5}) + B + D^\pm t$$

where $t = T/T_\mathrm{N} - 1$ is the reduced temperature. Initially, this left us with five free fit parameters for each fit. However, since the phononic contributions to c_p and α_i were already subtracted, both the offset B and the linear term $D^\pm$ were set to zero, leaving us with only three fit parameters. To further reduce the free parameters to two, we tried fixing $\alpha^\pm$, either to values from fits for $T < T_\mathrm{N}$ or to a value giving good fit results over a wide range, also beyond the actual fitting range. Our best least-square fitting results are presented in Tab. S3. For comparison results with fixed and free values for $\alpha^\pm$ are shown for $\alpha_\mathrm{a,mag}$ and $c_\mathrm{p,mag}$.

Table S3: Fit parameters for the critical scaling according to Eq. (5) in the main text. Unless indicated otherwise, $B = 0$ and $D = 0$ were fixed for all fits. The parameters $A^\pm$ are in units of 1/K and J/(mol K), for thermal expansion and specific heat, respectively. 'Range' gives the fitting range. '(f)' indicates that the quantity was fixed manually and not fitted.

	Range $T < T_\mathrm{N}$	α^-	A^-	E^-	Range $T > T_\mathrm{N}$	α^+	A^+	E^+	A^+/A^-
$\alpha_\mathrm{a,mag}$	$0.01 < \|t\| < 0.1$	0.088(7)	$7.7(4) \cdot 10^{-7}$	−1.77(3)	$0.01 < \|t\| < 0.1$	0.21(3)	$4.15(4) \cdot 10^{-7}$	−1.57(10)	0.54
					$0.01 < \|t\| < 0.1$	0.088 (f)	$3.04(5) \cdot 10^{-7}$	−2.01(5)	0.39
					$0.01 < \|t\| < 0.1$	0.116 (f)	$3.54(4) \cdot 10^{-7}$	−1.92(4)	0.46
$\alpha_\mathrm{b,mag}$	$0.08 < \|t\| < 0.1$	0.116 (f)	$-3.20(5) \cdot 10^{-7}$	−1.54(5)	$0.08 < \|t\| < 0.1$	0.11(2)	$-1.38(11) \cdot 10^{-7}$	−1.82(11)	0.43
$\alpha_\mathrm{c,mag}$	$0.006 < \|t\| < 0.06$	0.12(5)	$1.55(15) \cdot 10^{-7}$	−1.1(5)	$0.009 < \|t\| < 0.11$	0.23(4)	$6.7(3) \cdot 10^{-8}$	−0.9(3)	0.43
β_mag	$0.01 < \|t\| < 0.13$	0.11(1)	$7.2(3) \cdot 10^{-7}$	−1.59(3)	$0.01 < \|t\| < 0.13$	0.20(2)	$3.24(4) \cdot 10^{-7}$	−1.38(9)	0.45
$c_\mathrm{p,mag}$	$0.006 < \|t\| < 0.1$	0.162(2)	2.214(5)	−1.49(2)	$0.01 < \|t\| < 0.15$	0.21(2)	0.743(7)	−1.60(4)	0.34
	$0.006 < \|t\| < 0.1$	0.125 (f)	2.16(2)[a]	−2.06(5)	$0.01 < \|t\| < 0.15$	0.125 (f)	0.663(6)[b]	−2.06 (f)	0.31

[a] Also fitting D^-, with $D^- = 26(4)$ J/(mol K)
[b] Also fitting D^+, with $D^+ = 4.2(3)$ J/(mol K)

[1] A. Pring, B. M. Gatehouse, and W. D. Birch, American Mineralogist **75**, 1421 (1990).
[2] K. Momma and F. Izumi, Journal of Applied Crystallography **44**, 1272 (2011).
[3] K. H. Miller, P. W. Stephens, C. Martin, E. Constable, R. A. Lewis, H. Berger, G. L. Carr, and D. B. Tanner, Physical Review B **86**, 174104 (2012).
[4] E. Constable, S. Raymond, S. Petit, E. Ressouche, F. Bourdarot, J. Debray, M. Josse, O. Fabelo, H. Berger, S. de Brion, et al., Physical Review B **96**, 014413 (2017).
[5] Z. Wang, M. Schmidt, Y. Goncharov, V. Tsurkan, H.-A. Krug von Nidda, A. Loidl, and J. Deisenhofer, Physical Review B **86**, 174411 (2012).
[6] S. A. Nikolaev, V. V. Mazurenko, A. A. Tsirlin, and V. G. Mazurenko, Physical Review B **94**, 144412 (2016).
